# Supplementary material for: Are carbon emissions trading and green financial instruments synergistic? -Comprehensive quantitative research based on content analysis
Source: PLoS One. 2024 Mar 7;19(3):e0298601. doi: 10.1371/journal.pone.0298601 (PMC10919601; doi:10.1371/journal.pone.0298601)
Supplement: S1 Data — (ZIP) [file pone.0298601.s001.zip › Data/地级市Z_F工作报告.zip!/░▓╗╒/╗╞╔╜╩╨/└┤╫╘╣1⁄2╓┌║┼í╛┬φ┐╦╩2╛▌═°í┐_2017.docx]

各位代表：

　　现在，我代表市人民政府，向大会报告政府工作，请予审议，并请市政协委员和其他列席人员提出意见。

一、2016年工作回顾

　　过去的一年，是全面建成小康社会决胜阶段的开局之年。面对复杂多变的宏观环境和经济下行压力，全市上下在省委省政府和市委的坚强领导下，全面贯彻党的十八大和十八届三中、四中、五中、六中全会精神，深入学习贯彻习近平总书记系列重要讲话特别是视察安徽重要讲话精神，坚持稳中求进的工作总基调，牢固树立和践行新发展理念，扎实推进供给侧结构性改革，全力以赴稳增长、促改革、调结构、惠民生、防风险，整体经济保持了稳中有进、稳中向好的态势，完成了市六届人大六次会议确定的目标任务，实现了“十三五”良好开局。

**——主要指标全面增长**。预计全市实现生产总值576.8亿元、同比增长7.8%，财政收入99亿元、增长7%，固定资产投资597.7亿元、增长8.2%，社会消费品零售总额313.1亿元、增长11.4%，外贸进出口总额6.5亿美元、增长3.1%，上述五项指标增速分别比上年度提高1.7、4.5、7.2、0.4和33.8个百分点。

**——结构调整成效明显**。黄山现代服务业产业园入选省第二批战略性新兴产业集聚发展基地，战略性新兴产业产值170.2亿元、增长18.3%；工业对经济增长的贡献率达38.9%。全市服务业增加值295.2亿元、增长8.5%，服务业增加值占生产总值比重突破50%；三次产业比重为9.8：39：51.2。

**——基础设施建设取得突破**。我市历史上最大的水利枢纽工程——月潭水库建设全面拉开，全年完成投资8亿元。杭黄铁路建设全面提速，昌景黄和池黄高铁建设项目进入勘察设计阶段，综合交通枢纽加快形成。205国道（改建）和梅林南路主车道全线贯通，市经济开发区、现代服务业产业园和江南片区建设加快，中心城区“一环三片”发展格局初步形成。

**——人民生活持续改善**。城镇常住居民人均可支配收入28393元、增长8.3%，农村常住居民人均可支配收入12869元、增长8.4%。脱贫攻坚首战告捷，全市68个贫困村达到出列标准，20842人实现稳定脱贫。防汛抗洪抢险救灾全面胜利，在全省率先完成受灾群众水毁房屋重建工作。

　　一年来，主要做了以下工作：

**一是坚持聚焦发力、精准施策，经济保持平稳增长**。认真落实国家和省促进经济持续健康较快发展各项政策措施，及时出台推进供给侧结构性改革、降成本减轻实体经济企业负担、建设战略性新兴产业集聚发展基地等系列政策意见，实行市级领导联系重点项目、重点企业等制度，全年安排扶持实体经济发展财政资金4.32亿元，减轻企业负担4.98亿元，企业百元主营业务收入的制度性交易成本同比下降4.1%。全力扩大有效投入，组织开展重点项目“百日会战”，全年完成重点项目建设投资突破300亿元。泰达新材料首发上市报中国证监会，新增“新三板”挂牌企业3家、省股权托管交易中心挂牌企业22家，市内金融机构贷款余额609.1亿元、增长7.6%。

**二是坚持优化结构、着力转型，三次产业协调发展**。着力“做精一产、做强二产、做优三产”，加快产业转型升级。大力发展精致农业，全年实现农业增加值56.4亿元、同比增长2%，规上农产品加工企业产值208.6亿元、增长8.1%；新增特色产业基地3.6万亩，新建改造泉水鱼养殖池3260口；新发展生态高效茶园3.2万亩，茶叶一产产值突破30亿元。成功举办中国茶叶经济年会和中国休闲农业黄山峰会，我市被授予“中国名茶之都”称号，“太平猴魁茶”国家地理标志产品保护示范区获批。加快推进新型工业化进程，净增规上工业企业33家，实现规上工业增加值132.7亿元、增长10.2%。全市各类开发园区实现规上工业总产值574.6亿元、增长15.2%，实现税收19.5亿元、增长12%。全面实施“旅游+”战略，深入推进皖南国际文化旅游示范区和国家级旅游业改革创新先行区、全域旅游示范区建设，全年旅游接待量5187.1万人次、旅游总收入450.1亿元，分别增长11.2%和12.3%，其中，入境游客215.2万人次、增长10.3%，过夜登记游客855.8万人、增长6.9%。

**三是坚持改革创新、扩大开放，发展活力显著增强**。深入实施“放管服”改革，减少市级权责事项20项、涉企收费项目13项，探索建立公共服务清单和中介服务清单。深化商事制度改革，新增市场主体1.54万户、增长22.2%。在全国率先开展旅游管理领域相对集中行政处罚权试点。持续推进营改增扩围试点。市供销社转企改制取得实质性突破。国有资产和国有企业改革成效明显。全市统一规范开放的公共资源交易市场基本建成。不动产统一登记制度全面实施。国有林场改革扎实启动，集体土地确权登记颁证工作基本完成。优化科技创新环境，新增国家级创业基地1家、众创空间2家、星创天地2家、高新技术企业9家。发布黄山城市形象标识。承办中俄总理定期会晤委员会双方主席会晤、在华高端外国专家考察等重大活动，举办第二届联合国教科文组织名录遗产与可持续发展黄山对话会、黄山旅游节暨第五届安徽国际旅行商大会、“杭州G20畅游黄山”等活动，黄山海峡两岸交流基地获批设立。深化与德国斯特拉松市、瑞典瓦拉市等友好城市交往，推动与“一带一路”沿线国家的交流合作，黄山至曼谷包机成功首航，黄山航空口岸外国人签证业务正式启动，口岸出入境突破10万人次。持续推进招大引强，联合利华立顿茶叶生产基地、黄山全域旅游研究院有限公司等项目落户黄山，全年新签内资项目545个，到位资金170.3亿元、增长20%。

**四是坚持规划引领、建管并重，城乡面貌明显提升**。围绕打造现代国际旅游城市，加强城市设计，强化规划管控。中心城区建设步伐加快，黄山中路综合提升及人防地下空间开发利用、迎宾大道综合改造及地下管廊等项目快速推进，市生活垃圾综合处理厂开工建设，人行道及绿化提升和公共自行车租赁系统（一期）建成使用，黄山农产品物流园建成运营。启动编制黄山北站综合交通枢纽规划，歙黟一级公路建设提速，黄千高速开工前各项工作基本完成，芜黄、德上高速前期工作顺利推进。高度重视区县城、重点镇建设，完成12个特色小镇规划编制，宏村镇跻身首批中国特色小镇，全市户籍人口城镇化率31.8%，提高2个百分点。美丽乡村建设继续走在全省前列，歙县、黄山区获省美丽乡村建设先进县称号。市城市数据中心、社会服务管理信息化平台建成运行，成功申报全国电信普遍服务试点城市、宽带中国示范城市。在全省率先启动美丽社区建设。大力开展全域环境整治，全力争创全国文明城市。

**五是坚持绿色发展、传承保护，特色优势日益彰显**。黄山区、歙县、休宁县、黟县、祁门县获批国家重点生态功能区，黄山风景区入选首批国家绿色旅游示范基地。在2016年度长三角主要领导座谈会上皖浙两省签订新安江流域上下游横向生态补偿协议，补偿机制二轮试点深入实施，发起设立首期规模20亿元的新安江绿色发展基金。建成农药集中配送网点444个，配送农药实现零差价销售。太平湖国家重点支持生态环境保护专项全面完成，绩效考评全国第一。全面完成千万亩森林增长工程任务，大气环境质量稳居全省第一，地表水功能区和集中式饮用水水源地水质达标率分别达96%、100%，获批国家低碳城市试点。深入推进徽州古建筑保护利用工程，完成投资6.8亿元，皖南古村落保护协会成立，古民居产权规范流转试点列入省2016年深化改革经典案例。故宫博物院驻安徽黄山徽派传统工艺工作站、故宫学院徽州分院、故宫博物院博士后工作站挂牌成立，市经济开发区非遗创意产业园启动规划设计，徽州方言语音档案库建成，安徽中国徽州文化博物馆获评国家一级博物馆，为全省地级市首家。105个千万元以上重点文化产业项目完成投资81.2亿元，文化产业增加值增长12%。

**六是坚持以人为本、脱贫为先，民生福祉持续提升**。坚持财政支出重点保障民生，累计支出139.5亿元，增长8.1%。精心组织实施34项省市民生工程，完成投资43.9亿元。城镇新增就业2.1万人，城镇登记失业率3.68%。五项保险参保93.2万人次，城乡居民基本养老保险参保75.7万人。新农合参合率100%，参合人员补偿比进一步提升。机关事业单位、企业退休人员退休费人均月增加221元和148元，城市低保、农村低保、重点优抚和分散供养五保、集中供养五保补助标准分别提高11%、25%、10%和37%、52%，80周岁以上老人高龄补贴制度全面落实。发放城乡医疗救助金3286万元。实施21个棚户区改造项目，整治完成104个老旧小区。灵活就业人员纳入住房公积金制度范围，住房保障范围扩大到中等偏下收入家庭和在城镇稳定就业的外来务工人员。扎实推进地质灾害隐患点搬迁避让和工程治理， 924户、2766人消除地质灾害威胁。深入实施脱贫攻坚“十大工程”，认真开展“万名党员干部帮万户”“百企帮百村”，超额完成年度目标任务。

**七是坚持统筹兼顾、全面发展，社会保持和谐稳定**。全面落实市委市政府“人才10条”，引进高层次人才63名，新增省级博士后工作站1家。第二期学前教育三年行动计划完成，我市顺利通过义务教育发展基本均衡县（区）评估验收。继续调整优化中心城区教育布局，中华职校并入黄山职业技术学院，整合中职资源成立黄山炎培职业学校。深化医药卫生体制综合改革，市公立医院“三同步”改革全面完成。市二院、市疾控中心整体搬迁并投入使用。计划生育工作持续健康发展，人口自然增长率4.65‰。发行市民旅游一卡通年票。持证残疾人在市区范围内免费乘坐公交。成功举办首届全民健身运动会、黄山168商界精英徒步越野挑战赛，中国黄山国际登山大会升级为UTMB认证积分赛事,我市优秀运动员吕秀芝获第31届里约奥运会女子20公里竞走项目铜牌，实现我市奥运奖牌零的突破。全面落实安全生产责任制，各项指标均控制在省下达范围之内。成功创建省食品药品安全城市。加大矛盾纠纷排查化解力度，依法规范信访秩序，加强社会治安综合治理，社会大局保持和谐稳定。国防教育、国防动员、后备力量建设和军事设施保护、人民防空工作不断加强，荣获全国双拥模范城四连冠。妇女、未成年人、老年人、残疾人权益得到保障。新闻出版、广播电视、文学艺术、哲学社会科学、统计、地方志等工作得到加强。气象、地震、防灾减灾工作取得新进展，民族宗教、外事侨务、对台等工作取得新成绩。

**八是坚持依法行政、严实作风，自身建设不断加强**。扎实开展“两学一做”学习教育和“讲看齐、见行动”学习讨论，切实增强“四个意识”特别是核心意识、看齐意识。认真落实全面从严治党主体责任，严格遵守《关于新形势下党内政治生活的若干准则》《中国共产党党内监督条例》，全力抓好巡视和考核反馈意见整改。加强机关效能建设，组织实施政府提速工程，强化行风政风民主评议，推进政务公开和政府信息公开，“12345”统一呼叫中心上线运行，办理有效群众诉求1.2万余件，办结率100%。所有行政审批项目实现“一门受理、一门发证”，提前办结率达99.4%。扎实推进依法行政，健全完善重大事项依法决策机制，市政府相对集中行使行政复议权改革工作获“第四届中国法治政府奖”，颁布我市第一部政府规章——《规章制定和起草地方性法规程序规定》。自觉接受市人大及其常委会的法律监督、工作监督和市政协的民主监督，405件人大代表建议和政协提案全部办结。严格执行中央八项规定精神和省、市委有关要求，大力精简会议、文件和事务性活动，全市“三公”经费支出下降28.9%。认真落实党风廉政建设主体责任，加强廉政风险防控，强化行政监察和审计监督，深入开展侵害群众利益的不正之风和腐败问题专项整治，严肃查处了一批违纪违法案件。

各位代表！

　　过去的一年，我们能够顶住经济下行压力，克服诸多困难和不利因素，取得来之不易的成绩，这是省委省政府和市委坚强领导的结果，是市人大及其常委会和市政协监督支持的结果，是全市人民凝心聚力、真抓实干的结果。在此，我代表市人民政府，向全市人民，向各民主党派、各人民团体和各界人士，向驻黄解放军指战员、武警官兵和政法公安干警，向关心、支持黄山发展的中央、省驻黄单位和海内外友好人士，向在我市创业的投资者、建设者，表示衷心的感谢!

　　在肯定成绩的同时，我们必须清醒认识到存在的矛盾和问题。在经济发展上，发展不足、发展不优的问题仍很突出，实体经济困难增多，牵动性强的大项目特别是产业类项目不多，新的经济增长点尚未形成，财政收入质量不高，非住宅类商品房去化周期较长，经济回稳向好的基础还不稳固，转型升级任务仍然艰巨。在社会建设上，城乡居民收入增长的渠道不多，全面稳定脱贫的任务繁重，就业、社保、教育、医疗、养老等方面还存在诸多薄弱环节，城乡基础设施欠账较多，生态保护、防灾减灾、安全生产和社会治理的压力依然很大。在政府自身建设上，运用新理念引领新发展的能力有待提高，庸懒散拖等不作为现象还不同程度存在，服务理念、服务机制、服务水平还不能很好适应新形势新要求，一些领域腐败现象还时有发生。对此，我们一定高度重视，采取切实措施，认真加以解决，不辜负各位代表和全市人民的期望和重托。

二、2017年主要工作

　　今年是党的十九大召开之年，是实施“十三五”规划的重要一年，是供给侧结构性改革的深化之年，也是全面完成本届政府任期目标的收官之年，做好今年工作意义重大。一方面要看到，当前宏观经济形势错综复杂，区域发展竞争日趋激烈。另一方面也要看到，我国经济发展长期向好的基本面没有改变，国家和省将持续出台稳增长、促改革的系列政策措施，特别是把旅游业作为新常态下稳增长的新支点和重要支柱产业来培育，把生态文明建设摆上更加重要的位置，为我市当前及今后一个时期加快发展提供了有利的政策环境；随着我市综合交通体系的日趋完善，与长三角、京津冀、珠三角等城市群的时空距离大幅拉近，必将在迎接辐射中获取更多动能，为我们实施“十三五”规划和落实市第六次党代会确定的目标任务提供了广阔的发展空间。

　　根据市委六届二次全会精神，今年政府工作的总体要求是：全面贯彻党的十八大和十八届三中、四中、五中、六中全会精神，深入学习贯彻习近平总书记系列重要讲话特别是视察安徽重要讲话精神，认真落实省委省政府和市委决策部署，统筹推进“五位一体”总体布局，协调推进“四个全面”战略布局，坚持稳中求进工作总基调，牢固树立和贯彻落实新发展理念，适应把握引领经济发展新常态，以提高发展质量和效益为中心，以供给侧结构性改革为主线，以实施五大发展行动计划为总抓手，全力做好稳增长、促改革、调结构、惠民生、防风险各项工作，促进经济平稳健康发展和社会和谐稳定，以优异成绩迎接党的十九大胜利召开。

　　今年经济社会发展的主要预期目标是：全市生产总值增长8%左右，财政收入增长7%，固定资产投资增长8%，社会消费品零售总额增长10%，城镇常住居民人均可支配收入增长8.5%，农村常住居民人均可支配收入增长9%，居民消费价格涨幅控制在3%左右，万元生产总值能耗、主要污染物排放总量控制在省政府下达目标以内。上述目标安排体现了奋发有为、自我加压的工作要求，只要我们牢牢扭住发展第一要务，坚持“信心不可动摇、风险不可忽视”，凝心聚力，攻坚克难，就一定能够实现既定的目标任务。

　　积力之所举，则无不胜也；众智之所为，则无不成也。为此，我们将重点做好以下十个方面工作：

　　（一）以五大发展行动计划为引领，促进经济持续健康发展

　　坚持把稳增长作为经济工作的首要任务，深入推进供给侧结构性改革，巩固扩大经济稳定向好势头。

**全面实施五大发展行动计划**。加强统筹协调，强化战略谋划，加大政策支持，完善推进机制，认真落实省、市五大发展行动计划，扎实推进34项重点任务，组织实施50个重点工程，确保主要目标任务一年初见成效、三年实现突破、五年全面完成。通过五年的努力，完成重点项目投资3000亿元以上，力争地区生产总值突破1000亿元，人均主要经济指标走在全省前列，主要社会生态指标走在全国前列。

**深入推进“去降补”**。全面落实省“1+8+4”政策体系和市“1+N”促进产业发展政策，优化生产要素配置和组合，全面提高供给质量和效率。推进去产能，坚持做优增量、调整存量，分类处置僵尸企业，加大落后产能淘汰力度，推动传统产业向高新化、信息化转型升级。推进去库存，把去库存和促进人口城镇化结合起来，落实房地产去库存三年计划，提高棚改货币化安置和公租房货币化保障比例，积极促进房地产市场平稳健康发展。推进去杠杆，支持企业加大股权融资等直接融资力度，扩大政策性融资担保企业受益面，进一步降低企业杠杆率；规范政府举债行为，健全债务风险预警机制及应急处置机制，防范和化解政府债务风险。推进降成本，落实减税降费政策，加快企业电力直供，加大企业债券、专项债券、绿色债券争取力度，切实降低企业制度性交易、用能用地、物流等成本，力争工业企业主营业务成本降至全省平均水平。推进补短板，加大创新能力、产业发展、基础设施、公共服务等领域补短板力度，既补硬短板也补软短板，既补基础短板也补民生短板，切实增强发展的全面性、协调性、可持续性。

**全力扩大有效投入**。突出抓好300个以上市级重点项目，全年完成投资300亿元，新开工亿元以上项目70个、竣工30个以上。基础设施方面，推进杭黄铁路、宣城－黄山天然气干线、新安江街口跨江大桥建设，开工建设芜黄高速，争取开工建设黄千高速、德上高速、昌景黄高铁、池黄高铁，确保歙黟一级公路等项目建成运营，积极推进黄山北外环高速、歙县长陔岭隧道和屯溪国际机场迁建、旅游轻轨等重大项目前期工作。产业发展方面，加快推进“欧洲之星”欢乐谷、智谷科技园、神剑新材料三期、昌辉集团新能源汽车电子控制系统等项目建设，开工建设特拉斯智能充电设备、铁牛集团汽车灯具、博蓝特蓝宝石衬底片、黄山电器场效应管二期等项目，确保康师傅瓶装水、东晶电子等项目建成投产。这些重大项目的实施，必将为我市经济平稳健康发展注入强劲动力。

**着力提升项目工作水平**。抢抓中国制造2025、重大工程包、国家“三年滚动投资计划”等机遇，对接省五大发展行动计划和“三重一创”建设，谋划一批牵动性强的重大项目，建立五大发展项目库，力争更多项目进入国家和省年度投资计划。积极用好PPP项目建设模式，加大各类建设债券发行力度，拓宽社会资本参与基础设施和公共服务等投资领域。严格落实“四督四保”，建立健全目标责任、运行调度、督查考核等机制，全面提升项目工作水平。

　　（二）突出抓好工业转型升级，加快新型工业化进程

　　牢固树立“工业强市、产业兴市”理念，以《黄山市工业转型升级规划》为引领，着力壮大工业总量，优化产业结构，提升发展质量，全年规上工业增加值增长10%。

**培育壮大主导产业**。深入实施“个转企”“小升规”工程，全年净增规上工业企业30家以上。继续实施“市级队”培育计划，实现产值占规上工业比重提高2个百分点以上。大力实施百亿产业培育行动计划，组织实施绿色食品、汽车电子、绿色软包装、精细化工等四大主导产业发展规划，力争1～2个产业集群产值超百亿元。积极引进行业领军企业，实施水产业开发专项工程。抓好文化旅游省级战略性新兴产业集聚发展基地建设，启动建设装备制造、电子信息等市级战略性新兴产业集聚发展基地，战略性新兴产业产值增长12%以上。

**加快推进园区建设**。以百亿园区培育行动计划为抓手，聚焦主导产业和首位产业，建立健全园区初始企业孵化、信用担保、对接资本市场、精准帮扶等工作机制，加快“双清双控”和“腾笼换鸟”，力争清理低效用地1000亩以上，建成标准化厂房10万平方米以上，支持有条件的开发区建成一批加速器，全市各类开发园区工业增加值和固定资产投资均增长10%。创新市经济开发区管理体制，全面实行档案管理、全员聘用、绩效考核，开工建设未来科技城、小罐茶、菲英刹车总成等10个亿元以上项目，市经济开发区实现“去筹转正”。

**着力推进科技创新**。突出抓好86个重点工业项目调度，鼓励企业实施“零地技改”和“机器换人”，力争全年工业投资增长10%以上。深入开展“互联网+”制造行动，大力推进“两化融合”，力争建成300家智慧企业和一批数字化车间。支持企业开展产学研用合作，“市级队”企业、高新技术企业研发投入分别达到销售收入的2%、4.5%，新认定省级企业技术中心2家以上、省市工程技术研究中心8家，新组建院士工作站1家。推进国家火炬黄山软包装新材料特色产业基地和4个省级高新技术产业基地“二次创业”，净增国家高新技术企业6家，高新技术产业增加值增长12%。发扬“徽匠精神”，实施“三品”提升工程，积极争创驰名商标、著名商标。

**全力优化发展环境**。加大实体经济发展支持力度，加强政策落实，打通政策落地“最后一公里”。强化企业运行调度和协调服务，切实帮助企业解决生产经营中的困难和问题。大力发展民营经济，加强对各种所有制组织和自然人财产权保护，提升企业家信心，着力构建“亲”“清”新型政商关系，让每一位企业家专注创业、安心创业、愉快创业。

　　（三）积极推进“旅游+”，推动服务业大发展大提升

　　认真落实皖南国际文化旅游示范区“1+5”行动计划，大力发展以旅游业为重点的现代服务业，加快打造美丽中国先行区和世界一流旅游目的地，打响“中国旅游黄山再出发”品牌。

**全力推进旅游转型升级**。扎实推进国家级旅游业改革创新先行区建设，全力创建国家全域旅游示范区，力争游客接待量和入境游客均增长10%，旅游总收入增长12%。高标准抓好大黄山国家公园建设。完成黄山风景区世界生物圈保护区申报，通过全球可持续旅游目的地认证。加快黄山东部开发，力争东海景区详细规划六月底前通过国家审批，同步推进索道等综合配套设施项目前期工作。启动谭家桥国际小镇基础设施建设。抓好齐云山、牯牛降5A景区创建，推进太平湖国家级旅游度假区创建。提升屯溪老街、黎阳in巷、花山谜窟、新安江滨水旅游区品质，打造中心城区“旅游者空间”。大力发展摄影写生、户外运动、修学研学、宗族寻根等旅游新业态，加快建设一批特色旅游小镇（村），打造一批精品徽州民宿。加快推进“美丽之冠”、通用航空基地、“光大驿站”等重点旅游项目建设，持续打造黄山国际登山大会、黄山（黟县）国际山地车公开赛、黄山168国际徒步探险、国际摄影展会等国内外有影响力的品牌赛事活动。以举办建市30周年纪念活动为契机，创新办好黄山旅游节，持续加大“梦幻黄山礼仪徽州”城市品牌宣传力度。巩固长三角及省内近程市场，扩大高铁沿线等中远程市场。开通黄山至桂林等旅游航线，加密国际国内航班，巩固扩大香港和台湾、韩国及东南亚市场，积极开发欧美等新兴市场。加快黄山旅游大数据中心建设，抓好旅游管理领域相对集中行政处罚权试点，健全旅游综合执法体制机制，加强旅游应急救援力量建设，着力打造幸福旅游、美丽旅游、智慧旅游、信用旅游，实现黄山旅游发展再上新台阶。

**大力发展现代服务业**。加快做大黄山现代服务业产业园，以园区东部组团“大三角”区域开发为重点，加快基础设施建设，拉开园区框架；聚焦文旅主业，加大招商力度，开工建设中国黄山国际户外运动基地、黄山昌仁医养综合体等项目，建成创业小镇、金融小镇，力争完成投资20亿元，新开工亿元以上项目5个。加快省级服务业集聚区建设，促进规模化、集群化、网络化发展。发挥新消费引领作用，深入开展“十大扩消费行动”，大力发展现代金融、现代物流、文化创意、研发设计等生产性服务业，加快发展现代商贸、健康管理、养老养生等生活性服务业。力争服务业增加值增长不低于全市生产总值增幅，占全市生产总值比重提高1个百分点左右。

　　（四）扎实推进农业供给侧结构性改革，大力发展精致农业

以建设全省现代生态农业产业化示范市为抓手，加快推进农村一二三产业融合发展，着力提升现代农业发展水平。

**加快发展山区特色农业**。坚持特色化布局，扎实开展农业绿色增效行动，增加绿色优质农产品供给。大力实施茶产业发展行动计划，着力办好茶交会、黄山茶会等活动，提升黄山茶规模化、品牌化、清洁化发展水平，实现茶叶一产产值32亿元。加快林种结构调整，新发展以香榧、油茶、毛竹为主的特色高效经济林8000亩。抓好重点区域油菜示范片建设。扩大“一村一品”覆盖范围，因地制宜发展徽菊、干鲜果、食用菌、道地药材、皖南花猪、泉水鱼、黄山黑鸡等特色种养业。推进农业标准化生产，建立健全农产品质量安全可追溯机制，加快国家茶叶及农产品检测重点实验室建设，加强地理标志证明商标培育和管理，争创国家农产品质量安全示范市，让更多的黄山优质特色农产品成为市场青睐的绿色食品、安全食品。

**推动农村三次产业融合发展**。坚持以工业的理念发展现代农业，开展农业产业化攻坚行动，扶持壮大一批产值超亿元龙头企业、超10亿元产业化集群，新发展家庭农场150个，组建现代农业产业化联合体15个，力争规上农产品加工企业产值超过210亿元。实施休闲农业与乡村旅游提升工程，建设十大精致农业示范区，打造10个左右精品休闲农庄，推广复制“有农模式”，着力开发与休闲度假相融合的农业新业态、新产品，提升农业综合效益。深入开展电商黄山建设，支持淘宝、京东等在我市构建运营服务体系，全年农产品电子商务交易额6.5亿元，用互联网把小生产与大市场无缝对接起来，带动农业全产业链发展。

　　（五）全面深化改革扩大开放，激发经济社会发展活力

　　坚持把改革作为加快发展的根本动力，把开放作为繁荣发展的必由之路，让改革的红利加快释放，让开放的活力充分迸发。

**加快推动重点领域和关键环节改革**。深化“放管服”改革，动态调整完善权责清单、涉企收费清单，全面实施公共服务和中介服务清单，试行企业投资项目管理负面清单制度，实现“双随机、一公开”监管全覆盖。深化商事制度改革，积极推进“多证合一”。以赋予农民更多财产权利、增加财产性收入为目标，全面开展集体资产清产核资，有序推进农村集体产权制度改革。完成国有林场改革。深化林权配套制度改革，进一步做强江南林交所。探索实施公共资源交易领域相对集中行政处罚权。深化国企改革，支持黄山旅游集团“二次创业”，加快市城投集团转型发展，推动市文投、信保、供销集团改革任务落地，提升市交投公司交通基础设施建设融资功能。坚持企业创新、大众创业、政府创优同向发力，加强创业培训和孵化服务，打造一批众创空间、创新工场，培育一批创业团队和领军人物。

**全面扩大对外开放**。持续深化与杭州多层面合作，全面加强与长三角、京津冀、珠三角和海西经济区的对接与交流。坚持把招商引资引智作为重中之重，完善招商工作政策体系，健全项目预审、清单招商等工作机制，积极搭建重大活动、专题推介等精准招商平台，持续开展产业链招商、以商引商和驻外小分队招商，力争复星集团、山东鲁信、国药集团等项目早日落地，全年新签亿元以上项目130个，到位资金超过200亿元。拓展与国际组织、国际友城合作，加强与“一带一路”沿线国家的联系交往。加强机电等商品出口基地建设，进一步扩大茶叶出口欧美发达国家比重，保持外贸进出口稳定增长的良好势头。

　　（六）着力统筹城乡发展，加快建设现代化区域性中心城市

　　深入开展国家新型城镇化试点，扎实推进“五城联创”，积极争创全国文明城市、联合国人居环境奖城市，加快打造“第二居住地”。

**抓好中心城区和区县城建设**。全面完成205国道（改建）、梅林南路等项目建设，加快推进市经济开发区、黄山现代服务业产业园基础设施建设，建成地下综合管廊，完善江南片区服务配套功能，基本形成中心城区“一环三片”发展格局。建成黄山中路综合提升及人防地下空间开发利用项目，开工建设华山大桥，加快实施屯溪老街综合改造提升、花溪饭店地块改造、外边溪开发，构建三江口城市核心商圈。提升改造齐云大道延伸段等10条主次干道，贯通新园西路等5处道路节点，开展道路交口全面整治，提高道路通达性和路网完整性。完成人行道及绿化提升（二期）工程，加快公共停车场、全域租车项目、新能源汽车充电基础设施建设，深入开展电动车专项整治，争创省级优先发展公共交通示范城市。推进省级海绵城市试点建设，实施排水防涝“三年行动”，消除城区主要易涝点。建成市生活垃圾综合处理厂，开工污泥与餐厨垃圾处置项目。加快南部城镇群同城化步伐，开展城镇规划建设管理“六项行动”，推进老城区“双修”和新城区建设，积极打造特色街区，提高城市综合承载力。全年完成城镇固定资产投资500亿元以上，户籍人口城镇化率提高1个百分点。

**加快特色小镇建设**。抓好50个重点项目建设，力争完成投资30亿元，基本建成5个特色小镇。突出产业培育，主攻有基础和有优势的特色产业，加快形成完整的产业形态。引进战略投资者，充分运用PPP模式，吸引多元主体参与小镇建设，培育新的经济增长极和新型城镇化建设新平台。完善政策支持体系，设立特色小镇发展基金，建立健全动态调整、考核激励机制，探索黄山特色小镇经济发展新路。

**推进美丽乡村建设**。继续抓好2016年度美丽乡村建设任务，确保顺利通过省级验收。启动2017年度42个乡镇政府驻地建成区整治、46个省级中心村和20个左右市级中心村建设，推进世行贷款新农村建设项目，探索产村融合发展之路，打造一批精品村、特色村。逐步建立村庄保洁和公用设施长效管护机制，确保美丽乡村建设继续走在全省前列。

**提升城乡管理和公共服务水平**。编制市县域空间规划，加强中心城区和区县城重要节点的城市设计，实施城乡风貌环境整治。深化城管执法体制改革试点，建成全市数字城管一体化指挥平台。着力改善农村人居环境，启动农村生活污水综合治理项目，加快实施农村生活垃圾收运体系PPP项目，切实抓好“四好农村路”建设。深入实施城镇“十整治”、农村“六清理”行动，逐步实现全域环境整治市场化常态化长效化。切实加强市民文明素质养成教育，提升供水、供电、燃气、通讯等窗口单位服务水平。推进美丽社区建设，建成“三美四好”社区10个以上。逐步提高老旧小区物业管理覆盖面，让更多城市居民能够少些烦心、住得舒心。

　　（七）持续改善生态环境，永葆一方蓝天碧水

　　坚持绿水青山与金山银山有机统一，深入推进绿色发展、循环发展、低碳发展，争创国家生态文明试验区，着力打造美丽中国安徽样板的示范先行区。

**深入实施新安江流域综合治理**。以实施新安江流域生态补偿机制二轮试点为抓手，加快建设山水相济、人文共美的新安江生态经济示范区。抓好点面源污染防治、水资源保护、水生态修复等重点工作，推进畜禽养殖废弃物资源化利用体系建设，建成农药集中配送体系，完成流域城镇生活污水处理设施提标改造，争取实施山水林田湖生态修复工程，建立上下游联合保护区域合作机制，确保水质稳定向好。加快月潭水库建设，确保汛期前完成大坝主体工程，全年完成投资10亿元以上。用好新安江绿色发展基金，探索建立产业准入负面清单和多元化补偿机制。

**切实加强生态保护和环境治理**。突出抓好资源节约集约利用，全面落实能耗、水资源消耗、建设用地总量和强度双控目标，加快建设覆盖全市的水资源管理监控系统和监管体系，提高土地资源利用效率。扎实开展林业增绿增效行动，全年新造林2万亩。深入开展阻击松材线虫病专项行动，确保森林资源安全。全面推行河长制，积极争取太平湖流域国家第二轮水环境保护专项和以阊江流域治理为重点的水生态环保项目，加快实施中小河流治理、水土保持、“小农水”等项目。加强地质灾害防治，完成30处以上地质灾害隐患点治理任务。推进大气、水、土壤污染防治，实施重点行业污染源全面达标排放计划。认真执行生态文明建设目标评价考核办法，严格落实生态环境损害责任终身追究和赔偿制度，确保主要生态指标保持全国一流水平，让黄山青山常在、绿水常流、空气常新。

　　（八）坚持传承与创新并举，繁荣发展徽州文化

　　大力弘扬徽州文化，推动徽州文化创造性转化、创新性发展，全力打造中国优秀传统文化传承创新区。

**传承保护徽州文化**。深入实施徽州古建筑保护利用工程，推进徽派建筑数据库建设，完善古建筑保护利用和产权流转法律体系，继续对4种空间形态、12种单体形态古建筑实施保护，实施160个以上重点保护利用项目。组织申报第五批中国传统村落，加快古建筑群消防安防工程建设，支持歙县古城墙申报世界文化遗产，力争黟县获批国家历史文化名城。深入推进徽州文化生态保护实验区建设，加快设立徽州书院，实施名镇名村名街志编纂工程，出版发行《徽州文物谱系》，完成全市可移动文物普查。举办第三届中国非遗传统技艺大展。用好市政府与故宫博物院建立的合作机制和平台，争取创办黄山市行知非遗技艺学院，抓好国家级非遗项目、传习基地和传承人队伍建设，推进徽州四雕、徽墨歙砚、万安罗盘、徽州漆器等保护性开发，让传统技艺走进现代生活、现代设计走进传统工艺，推动徽州非遗经济的良性发展。

**扩大公共文化服务**。开工建设黄山市文化艺术中心和歙县徽州历史博物馆，建成开放市城市展示馆、市摄影博物馆，力争市图书馆跻身国家一级馆。积极开展公共文化服务标准化、均等化试点，推动公共文化服务向社区和农村基层延伸。实施文化惠民工程，全面实行公共文化设施免费开放，广泛开展群众性文化活动，加快建设书香黄山，实现物质富足与精神富有相统一。

**加快发展文化产业**。抓好100个重点文化产业项目建设，完成投资75亿元，力争文化产业增加值增长12%。加快文化产业集聚区建设，支持重点文化企业做大做强，争创省级以上文化产业示范基地1～2家。用好省股权交易托管中心文旅专板，推动文化企业挂牌上市。开工建设市经济开发区非遗创意产业园，积极争创国家级文化产业示范园区，努力打造徽州文化产业示范窗口和亮丽名片。

　　（九）扎实做好财政金融工作，增强发展保障能力

　　进一步优化财政收支结构，强化金融支持保障，全面提升财政金融服务水平。

**规范财政运行管理**。全面落实结构性减税政策和产业扶持政策，建立完善竞争性分配机制，提升财政资金扶持重点产业和重点企业的效果。完善综合治税措施，推动国地税征管体制改革，依法加强税收征管，优化收入结构，提高收入质量。理顺市和经济开发区、屯溪区财政体制，合理划分财政事权和支出责任。完善全口径预算编制，加强国有资本经营预算管理，强化支出绩效评价，建立政府投资项目预算评审制度，提高政府资金使用效益。深化国有资产管理改革，着力抓好市场化运营，确保国有资产保值增值。

**强化金融服务工作**。加大金融服务实体经济特别是中小微企业力度，引导金融机构持续扩大信贷总量，力争全市金融机构贷款余额突破650亿元，为企业解决“过桥贷款”资金突破35亿元。支持金融机构创新金融产品，力争“税融通”贷款突破4亿元、政银担合作业务突破33亿元。加快多层次资本市场体系建设，积极推进泰达新材料、科宏生物主板上市进程，建立完善企业上市储备制度，推动利用企业债、公司债、中期票据、短期融资券等债务融资，力争全年新增“新三板”挂牌上市企业3家，直接融资45亿元。充分发挥安元现代服务业等基金的投融资作用，积极推动设立市产业发展引导基金。深入实施金融扶贫工程，用好建档立卡贫困户小额贷款政策。大力发展普惠金融，稳妥推进农村资金互助，做好黄山区全国农村承包土地经营权抵押贷款试点工作。加强金融市场监管，严厉打击各类非法集资行为，有效防范和化解金融风险，构建黄山良好的金融安全生态。

　　（十）织密扎牢民生保障网，促进社会和谐稳定

　　集中精力做好普惠性、基础性、兜底性民生建设，努力建设更加稳定、和谐、有序的平安黄山。

**扎实推进精准扶贫精准脱贫**。严把识别、施策、帮扶、脱贫“四个关口”，深入实施脱贫攻坚“十大工程”，加快推进精准扶贫、监测评估、防范返贫、责任落实“四个机制”全覆盖。按照“五个更加”的要求，强力推进基础设施扶贫，加快改善贫困地区生产生活条件，确保完成年度易地扶贫搬迁任务；强力推进产业扶贫，大力发展“龙头企业+合作社+贫困户”生产经营模式，确保每个贫困村至少发展1项特色产业、每个有劳动能力的贫困家庭至少能从事1项特色种养业；强力推进健康扶贫，认真落实健康脱贫“351”政策，加快构建覆盖所有贫困人口的医疗保险和救助制度；强力推进兜底扶贫，落实农村低保标准和扶贫标准“两线合一”，全面加强特殊群体帮扶工作；强力推进合力扶贫，深入开展“万名党员干部帮万户”“百企帮百村”，力争全市超2万人脱贫、85个贫困村出列，确保小康路上不落一户、不掉一人。

**切实做好民生保障工作**。认真组织实施好省市民生工程，健全常态长效管养机制。加快城镇棚户区改造和老旧小区整治,确保全年完成50个、6954户棚户区改造和50个老旧小区整治任务。推进高校毕业生等重点群体就业，城镇新增就业2万人，城镇登记失业率控制在4.2%以内。实施全民参保登记计划，五项保险参保93万人次，城乡居民基本养老保险参保75.8万人。提高新农合、城镇居民医保、基本公共卫生服务财政补助标准，实施重度残疾人护理补贴，加强对特定人群特殊困难的帮扶。推广医养结合养老模式，支持社会力量兴办养老服务机构，提升农村敬老院服务质量和水平，让全市人民有更多获得感。

**大力发展各项社会事业**。深入实施人才优先发展战略，引进高层次人才30名，新增博士后工作站1家。启动实施第三期学前教育三年行动计划，推动义务教育优质均衡，有序推进普通高中课程和考试评价制度改革。加快职业教育资源市级统筹步伐，支持黄山职业技术学院现代旅游业、现代服务业等高技能人才培养，积极推进国际护理学院筹建工作。支持黄山学院建设有特色、高水平的应用型大学。加快健康黄山建设，深入推进医药卫生体制综合改革，完善公立医院法人治理结构和治理机制，深化人事薪酬制度改革，加快推进分级诊疗，继续推进“三名”工程建设，进一步打响新安医学特色品牌。广泛开展全民健身运动，举办第七届市运会。深入实施全面两孩政策，加大计生困难家庭帮扶力度。做好第三次全国农业普查。评选表彰第六届黄山市劳动模范和先进工作者。支持工会、共青团、妇联等人民团体依据法规和章程开展工作。切实维护妇女、未成年人、老年人、残疾人合法权益。做好民族、宗教等工作。推动军民融合深度发展，加强国防教育、国防动员、后备力量建设和军事设施保护、人民防空工作，争创全省双拥模范城七连冠。

**加强和创新社会治理**。推广应用社会服务管理信息化平台，完善“12345”统一呼叫中心功能，提高网格化管理和一站式服务水平。加强农村基层组织建设，完成第十届村委会换届选举。完善政府购买服务机制，发挥社会组织在社会治理和公共服务中的积极作用。深入开展“七五”普法。深化信访工作制度改革，健全完善多元化矛盾纠纷化解机制，发挥“两个中心、两个工作站”作用，努力从源头上防范化解社会矛盾。坚持党政同责、一岗双责、齐抓共管、失职追责，深化“铸安行动”，加强重点行业领域安全专项治理，严格排查和整治各类安全隐患，确保各项安全生产指标控制在省下达范围之内。严防、严管、严控食品安全风险，积极争创国家食品安全示范城市。推进应急体系建设，突出抓好消防安全，切实做好防汛抗旱、森林防火和气象、地震等防灾减灾工作。构建立体化数字化社会治安防控体系，推进公共安全视频大数据项目建设，加强网络信息安全管理，依法打击各类违法犯罪行为，确保社会大局和谐稳定。

三、加强政府自身建设

　　决战决胜全面小康，建设更美丽更富裕更文明的黄山，我们承载着更大期盼，肩负着更大责任。政府全体工作人员必须坚定理想、坚定信念、坚定看齐，将“忠诚、干净、担当”内化于心、外化于行，认真践行“三严三实”，大力推进服务创新，着力打造为民务实高效清廉的政府。

**一要讲忠诚严要求，全面推进从严治政**。巩固拓展“两学一做”学习教育和“讲看齐、见行动”学习讨论成果，深入学习贯彻习近平总书记系列重要讲话特别是视察安徽重要讲话精神，牢固树立“四个意识”特别是核心意识、看齐意识，自觉在思想上政治上行动上同以习近平同志为核心的党中央保持高度一致。坚持在全局下定位、大局下行动，坚决贯彻落实省委省政府和市委的重大决策部署，确保政令畅通、令行禁止。把纪律和规矩挺在前面，严格落实《关于新形势下党内政治生活的若干准则》《中国共产党党内监督条例》，坚持党的组织生活各项制度，严格执行民主集中制，努力营造风清气正的政治生态。

**二要讲法治强规范，全面推进依法行政**。认真履行法治政府建设职责，严格按照法定权限和程序行使权力。推进重大决策合法性审查全覆盖，严格执行各项法定程序，进一步规范政府重大事项决策行为。加强政府立法工作，出台城市市容和环境卫生管理规定、黄山风景名胜区管理条例实施办法，认真做好徽州古建筑保护利用条例、松材线虫病防治条例起草工作。推进依法行政示范单位创建，严格规范行政执法行为，深化政府相对集中行使行政复议权改革，加强政府工作人员法制教育，强化执法监督，不断提升依法行政工作水平。深入推进政务公开标准化建设，加强政府网站、政务微博、政务微信等平台建设，建立完善第三方测评、网民测评反馈等督查考评机制。坚持重大决策出台前向人大及其常委会报告制度，加强与政协民主协商，广泛听取各民主党派、工商联和无党派人士意见，自觉接受人大及其常委会的法律监督、工作监督和政协的民主监督，认真落实人大及其常委会的决议决定和审议意见，认真办理人大代表议案、建议和政协提案，提高办理质量和满意度。

**三要讲担当重落实，全面推进高效施政**。持续推进机关效能建设，组织实施政府服务创新工程，实现政府服务提质、效能提速、工作提效。推进政府工作项目化、责任化，强化绩效管理，完善考核体系，加大督查问责力度，确保各项决策部署落地生根。健全落实干部关爱激励机制和容错纠错机制，为改革创新者撑腰鼓劲，让广大干部愿干事、敢干事、能干事、干成事。加强政务中心服务标准化建设，开展“减证便民”专项行动，积极推广“互联网+”政务服务，优化完善网上办事大厅功能，实现由“群众跑腿”向“数字跑路”转变。

**四要讲清廉守底线，全面推进廉洁从政**。认真贯彻《中国共产党廉洁自律准则》《中国共产党纪律处分条例》，严格落实党风廉政建设主体责任和“一岗双责”，深入推进政府系统党风廉政建设和反腐败斗争。严格执行中央八项规定精神和省、市委有关要求，巩固扩大巡视反馈问题整改成果，推进作风建设常态化、长效化。全面推行痕迹管理，加强对重点领域和关键环节权力运行的制约监督，进一步规范项目招投标、土地使用权与矿业权出让、国有产权交易、政府采购等行为。健全廉政风险防控机制，完善行政权力电子监察平台建设，推进审计监督全覆盖。始终保持惩治腐败的高压态势，扎实开展突出问题专项整治，严肃查处侵害群众利益的不正之风和腐败问题，以政府的“清廉指数”提升人民的“满意指数”。

　　各位代表！

　　处在百舸争流的发展时代，站在不进则退的历史潮头，唯有奋力争先、奋勇向前，方能不辱使命、不负重托。让我们紧密团结在以习近平同志为核心的党中央周围，在省委省政府和市委的坚强领导下，凝心聚力、真抓实干，不忘初心、继续前进，为把黄山建设得更美丽更富裕更文明、夺取全面建成小康社会伟大胜利而努力奋斗！
